# Predictors of Memory Deficits in Adolescents and Young Adults with Congenital Heart Disease Compared to Healthy Controls

**DOI:** 10.3389/fped.2016.00117

**Published:** 2016-10-31

**Authors:** Nancy A. Pike, Mary A. Woo, Marie K. Poulsen, Wendy Evangelista, Dylan Faire, Nancy J. Halnon, Alan B. Lewis, Rajesh Kumar

**Affiliations:** ^1^School of Nursing, University of California Los Angeles, Los Angeles, CA, USA; ^2^Division of General Pediatrics, Children’s Hospital Los Angeles, Los Angeles, CA, USA; ^3^Division of Pediatric Cardiology, University of California Los Angeles, Los Angeles, CA, USA; ^4^Division of Pediatric Cardiology, Children’s Hospital Los Angeles, Los Angeles, CA, USA; ^5^Department of Anesthesiology, University of California Los Angeles, Los Angeles, CA, USA; ^6^Department of Radiological Sciences, University of California Los Angeles, Los Angeles, CA, USA; ^7^Department of Bioengineering, University of California Los Angeles, Los Angeles, CA, USA; ^8^The Brain Research Institute, University of California Los Angeles, Los Angeles, CA, USA

**Keywords:** memory, working memory, congenital heart disease, anxiety, self-efficacy

## Abstract

**Introduction:**

Adolescents and young adults with congenital heart disease (CHD) show a range of memory deficits, which can dramatically impact their clinical outcomes and quality of life. However, few studies have identified predictors of these memory changes. The purpose of this investigation was to identify predictors of memory deficits in adolescents and young adults with CHD after surgical palliation compared to healthy controls.

**Method:**

One hundred fifty-six adolescents and young adults (80 CHD and 76 controls; age 14–21 years) were recruited and administered an instrument to assess memory [Wide Range Assessment of Memory and Learning Second Edition – general memory index (GMI) score] and completed questionnaires that measure anxiety, depression, sleepiness, health status, and self-efficacy. Descriptive and non-parametric statistics were used to assess group differences, and logistic regression to identify predictors of memory deficits.

**Results:**

CHD subjects consisted of 58% males, median age 17 years, 43% Hispanic, and medians of 2 previous heart surgeries and 14 years since last surgery. Memory deficits (GMI ≤ 85) were identified in 50% CHD compared to 4% healthy controls (median GMI 85 vs. 104, *p* < 0.001). Of GMI subscale medians, CHD subjects had significantly worse memory performance vs. healthy controls (verbal 88 vs. 105, *p* < 0.001; attention 88 vs. 109, *p* < 0.001; working memory 86 vs. 108, *p* < 0.001). No significant differences appeared between groups for visual memory. Multiple clinical and psychosocial factors were identified which were statistically different on bivariate analyses between the subjects with and without memory deficits. By multivariate analysis, male gender, number of surgeries, anxiety, and self-efficacy emerged as independent predictors of memory deficits.

**Conclusion:**

Adolescents and young adults with CHD, more than a decade since their last surgery, show significant verbal, attention, and working memory deficits over controls. To enhance patient memory/self-care, clinicians should explore ways to reduce anxiety, improve self-efficacy, and increase use of visual patient education material, especially in CHD males.

## Introduction

Memory is an important part of cognition and is inter-related with executive function skills. Memory deficits have been reported in up to 57% of adolescents with congenital heart disease (CHD) who have undergone surgical palliation (1–5). The mechanisms contributing to memory or neurocognitive deficits are multifactorial and are likely to include factors that are related to the CHD, including cyanosis, early cardiac surgery, a wide range of genetic syndromes or gene mutations, and prenatal and other pre- and postoperative factors that can have significant adverse effects on brain development and/or injury (6–9). However, these deficits may not become apparent until school-age, when higher-level organizational skills are required and can be especially problematic in adolescents who must eventually take responsibility for their health during the transition to adulthood. Furthermore, memory deficits can significantly impact the adolescent’s ability to name and follow a prescribed medication regime, adhere to preventative care and appointments, and potentially impact educational achievement, employability, and quality of life.

The improved survival of infants with complex CHD has been attributed to advancements in fetal detection, improved surgical techniques, and perioperative care (10) but many are at risk for neurodevelopmental and cognitive delays (8, 9, 11–13). Interestingly, risk factors or predictors of cognitive deficits have been more associated with innate- or patient-related factors (e.g., genetic syndrome, prematurity or weeks gestation, socioeconomic status, and maternal education) vs. intraoperative or postoperative management strategies [e.g., duration of cardiopulmonary bypass (CPB), hospital length of stay] (8, 9, 11–14). Memory deficits have emerged in some adolescent neuropsychological studies with worse memory identified in more severe or complex CHD (1–3). However, these studies lack the investigation of other potential patient or behavioral-related factors that could affect or produce transient memory loss (e.g., anxiety, depression, attentional disorders, excessive sleepiness, low self-efficacy, and perceived health status) (15–18). It remains unclear to what extent these memory deficits at a younger age persist to adolescence and young adulthood. Currently, no studies have specifically focused on predictors of memory deficits in adolescents and young adults with CHD. The identification of modifiable variables could potentially improve memory and subsequently the ability for self-care. Therefore, our specific aim of this study was to identify predictors (including clinical and behavioral factors) of memory deficit in adolescents and young adults with CHD after surgical palliation compared to age- and gender-matched healthy controls.

## Materials and Methods

### Study Population and Design

This is a cross-sectional, comparative study of 156 adolescents and young adults (80 CHD and 76 Healthy) recruited *via* flyers or provider referrals from University of California Los Angeles (UCLA) and Children’s Hospital Los Angeles (CHLA) pediatric cardiology clinics and non-hospital based cardiology clinics in Southern California. This study was carried out with Institutional Review Board approval from both UCLA and CHLA. We included adolescents and young adults with CHD between the age of 14 and 21 years, who have undergone surgical palliation requiring CPB. CHD participants were excluded, if they had isolated coarctation of the aorta or patent ductus arteriosus (not requiring CPB), previous head injury (e.g., concussion, stroke) severe developmental delay precluding active study participation and self-reporting (e.g., cerebral palsy, severe hypoxic-injury, or genetic syndrome associated with cognitive delays). If eligible, either a same day or future appointment was made to participate in the study.

Healthy controls were recruited from local high schools and community flyer. This control group was recruited instead of using normative data to capture the high prevalence of Hispanic ethnicity in the City of Los Angeles. Participants were screened by self-report and excluded for any chronic medical or psychiatric conditions or any previous head injury. If eligible, controls were matched to a CHD participant for age (±2 years) and gender, and an appointment was made to participate in the study either at their home, public library, or research office.

### Procedure

After parental permission and assent were obtained from participants under age 18, and informed consent was obtained from participants aged 18 and over, all study procedures were performed with the adolescent and test administrator in a private room. The test administrators were two trained graduate research assistants who met qualifications for administration with interrater agreement of 98% for the Wide Range of Assessment Memory and Learning second edition (WRAML2). The WRAML2 core and optional subtests were completed on all subjects (approximately 1 h) followed by self-administered questionnaires (approximately 15–20 min). Demographic and clinical data were obtained from a limited medical record review which included age, gender, ethnicity, type of CHD, number and type of surgical procedures, first surgery performed <30 days of life, cyanosis at birth, presence of genetic syndrome, attention deficit disorder (ADD) and/or hyperactivity (ADHD), and use of remedial educational services.

### Memory

Memory was measured using the WRAML2. This administered test is a highly reliable and valid measure of memory and learning abilities in subjects from age 5 to 90 years (19). The broad age range of the WRAML2 was important in instrument selection because of the need to cover adolescents and young adults compared to using two different measures (i.e., child and adult). The WRAML2 is a comprehensive test that measures an overview of memory function which consists of verbal and visual memory, attention/concentration, working memory, and memory recognition. The core battery consists of six subtests [story memory, verbal learning, design memory, picture memory, finger window (short-term memory of a visual sequential pattern), numbers/letters (digit-span format using both numbers and letters)] that when combined yield a general memory index (GMI) score (mean 100, SD ± 15) (19). Additional optional subtests performed were working memory and memory recognition yielding the general memory recognition index (GMR) score (mean 100, SD ± 15). The GMI measures immediate recall and the GMR measures delayed recall. The WRAML2 or GMI has been used in previous cognitive studies in children with and without CHD (20–22). The alpha reliabilities for the core subtests range from 0.85 to 0.94 (GMI 0.93) (19).

### Perceived Health Status

Perceived health status was measured using the Short-Form-36 Health Survey Version 2 (SF-36v2). The SF-36 was originally developed to measure perceived health status. However, many researchers incorrectly use the terms *health-related quality of life* or *quality of life* in reference to what the SF-36 measures (23). This self-reported questionnaire consisting of 36 Likert questions with 8 health concepts that assess perceived health status in: (1) physical function, (2) the physical function limitation as result of physical injury, (3) the role due to emotional problems, (4) energy and vitality, (5) mental health, (6) social function, (7) physical pain, and (8) general health (24). Two summary scores are calculated from the eight domain scores which include a physical component summary (PCS) and mental component summary (MCS). Summary scores range from 0 to 100 with higher scores indicating better health status (24). The SF-36 has been used in other studies in CHD (18, 25–27). Correlations between the SF-36 and other health status measures ranged from 0.51 to 0.82 in mental health, and 0.52 to 0.85 in physical health (24).

### Anxiety

Anxiety levels were measured using the Beck anxiety inventory (BAI) (28). The self-reported BAI is a 21-item questionnaire that measures the severity of common anxiety symptoms. Responses are rated on a 4-point (0–3) Likert-type scale (0 = “not at all bothered” to 3 = “severely bothered”) with a scores ranging from 0 to 63. Higher scores indicate greater anxiety severity (0–21 as low anxiety to 36–63 as severe anxiety). The BAI has been used in previous studies in CHD with a Cronbach’s alpha of 0.93 (29).

### Depression

Depressive symptoms were measured using the Patient Health Questionnaire Depression Module (PHQ-9). The self-reported PHQ-9 is a 9-items questionnaire, responses are rated on a 4-point (0–3) Likert-type scale (0 = “not at all bothered” to 3 = “bothered nearly every day”), with scores ranging from 0 to 27. Higher scores indicate greater depression severity (0–4 = no depression to greater than 20 = severe depression) (30). The PHQ-9 is a widely used measure of depression severity in the CHD population with a Cronbach’s alpha range of 0.86–0.89 (18, 31).

### Sleepiness

Excessive sleepiness was assessed using the Epworth sleepiness scale (ESS) (32). The self-reported ESS is a 9-item questionnaire, responses are rated on a 4-point (0–3) Likert scale (0 = “no chance of falling asleep” to 3 = “high chance of falling asleep”), with scores ranging from 0 to 24. Higher scores on the ESS indicate more excessive sleepiness (≥10 is considered positive). The ESS is a widely used instrument with established reliability and validity with a Cronbach’s alpha of 0.86 (32).

### Self-Efficacy

Self-efficacy was measured using the general self-efficacy (GSE) scale. The GSE is a 10-item questionnaire used to assess the belief in one’s own ability to cope with difficult demands in life (33). The self-reported responses are rated on how true the statement is for the person on a 4-point Likert-type scale (1 = “not at all true” to 4 = “exactly true”) with scores ranging from 10 to 40. Higher scores indicate greater self-efficacy with a Cronbach’s alpha range from 0.76 to 0.90 (33).

### Statistical Analysis

Characteristics of the sample are presented as means with SD or medians with range for continuous variables. Subjects were classified into two groups (CHD and Healthy). Variables were examined for normality and outliers. The continuous data had non-normal distributions (per Shapiro–Wilks tests of normality), and groups were compared using non-parametric statistics consisting of the Mann–Whitney *U* test for all continuous variables and Chi-squared for all categorical variables. Spearman’s rho correlation coefficients between all predictor and the outcome variable GMI were examined. Only predictors with significant correlations (*p* < 0.05) to the outcome variable GMI were entered into the multivariable analysis. The stepwise logistic regression model was performed for the binary variable of GMI ≤85 and >85 (1 SD below the expected population mean 100). The software identified the sequence of entry of covariates into the statistical model. All analyses were conducted using the Statistical Package for the Social Sciences version 23.0 (IBM; Somers, NY, USA).

## Results

### Sample Characteristics

Demographic characteristics of the CHD and healthy control groups are summarized in Table 1. No statistically significant differences in age, gender, ethnicity, and education emerged between groups. However, ethnicity showed a trend toward significance (*p* = 0.06) with 41 and 39% Hispanic ethnicity in the CHD and control group, respectively. Public insurance was higher in the CHD group (68%) compared to controls (10%) and the incidence of having ADD or ADHD (21%) compared to controls (4%). Use of remedial education services was identified in 24% of the CHD group only. Clinical characteristics of the CHD group are summarized in Table 2. Fifty percent of the sample had complex CHD, first surgery <30 days of age (58%), median of 2 previous surgeries, median 14 years from last surgery, taking 1–2 medication (48%), and New York Heart Association class I (64%).

**Table 1:** **Demographic characteristics between the congenital heart disease and control groups**.

Variable	CHD (*n* = 80)	Healthy (*n* = 76)	*p*-Value
Age (median)	17 (14–21)	18 (14–21)	0.700
Gender %			1
Male	42 (58%)	42 (58%)	
Female	38 (42%)	34 (42%)	
Ethnicity %			0.066
White	35 (44%)	36 (47%)	
Hispanic	33 (41%)	30 (39%)	
Other	12 (15%)	10 (14%)	
Education %			0.410
9th–11th grade	49 (61%)	47 (62%)	
High school graduate	18 (22%)	15 (20%)	
1–4 years of college	13 (13%)	14 (18%)	
Insurance^a^			<0.001*
Private	16 (20%)	38 (50%)	
Public	54 (68%)	8 (10%)	
Unknown/self pay	10 (12%)	30 (40%)	
ADHD/ADD %	17 (21%)	3 (4%)	<0.001*
Remedial education %	19 (24%)	0 (0%)	<0.001*

*ADHD, attention deficit hyperactivity disorder; ADD, attention deficit disorder; CHD, congenital heart disease; CPB, cardiopulmonary bypass; N/A, not applicable*.

*^a^Insurance was self-reported in healthy controls*.

*Statistically significant (*p* < 0.05).

**Table 2:** **Clinical characteristics of the congenital heart disease group**.

Clinical variables	*n* = 80 (%)
Defect severity^a^	
Simple	4 (5%)
• Isolated atrial septal defect (ASD)	2
• Isolated ventricular septal defect (VSD)	2
Moderate	36 (45%)
• Tetralogy of Fallot with or without pulmonary atresia (PA)	14
• Aortic stenosis	11
• Coarctation of the aorta (COA) with VSD and/or ASD	5
• Mitral stenosis/regurgitation	2
• Ebstein anomaly	2
• Partial atrioventricular canal (AVC)	1
• ALCAPA	1
Great complexity	40 (50%)
• Transposition of the great arteries (d-TGA)	14
• Congenitally corrected TGA (s/p double switch)	2
• Truncus arteriosus	1
• PA with intact ventricular septum (IVS)	1
• Single ventricle (s/p modified Fontan procedure) (*n* = 22)	
○ HLHS, DORV, unbalanced AVC (single right)	12
○ TA, DILV, unbalanced AVC (single left)	10
First surgery <30 days of age	46 (58%)
# Surgeries with CPB (median)	2 (1–5)
Years since last surgery (median)	14 (1–20)
Genetic syndrome %	2 (4%)
# Medications	
None	15 (19%)
1–2	38 (47%)
3–4	20 (25%)
≥5	7 (9%)
Pacemaker	10 (11%)
Cyanosis at birth	48 (60%)
Current oxygen saturation <90%	9 (12%)
NYHA classification	
I	51 (64%)
II–III	29 (36%)

*^a^Based on Bethesda conference classification*.

*HLHS, hypoplastic left heart syndrome; DORV, double outlet right ventricle; TA, tricuspid atresia; DILV, double inlet left ventricle; ALCAPA, anomalous left coronary artery to pulmonary artery; NYHA, New York Heart Association*.

### Memory, Anxiety, Depression, Sleep, Self-Efficacy, and Health Status Scores between Groups

Comparison of memory, anxiety, depression, sleepiness, self-efficacy, and health status between CHD and healthy controls is summarized in Table 3. In the CHD group, 50% scored 1 SD below the normal for GMI and 8% were 2 SD below the normative of 100 compared to 4 and 0% in the controls, respectively. Furthermore, GMI scores 1 SD below the normal were greater in males [*n* = 26 (62%)] vs. females [*n* = 9 (32%)]. Median GMI scores between CHD and healthy controls were 85 vs. 108, *p* < 0.001, respectively. All subgroups of the GMI and GRI showed statistically significant differences between CHD and controls except for visual memory.

**Table 3:** **Comparison of memory, anxiety, depression, sleepiness, self-efficacy, and health status between congenital heart disease and healthy control groups**.

Variables	CHD (*n* = 80)	Healthy (*n* = 80)	*p*-Value
		
	Median (range)		
WRAML2			
General memory index	85 (49–112)	108 (85–123)	<0.001*
Visual memory	100 (79–115)	100 (79–127)	0.162
Verbal memory	88 (69–120)	105 (80–135)	<0.001*
Attention/concentration	88 (37–117)	109 (65–134)	<0.001*
General recognition index	93 (57–122)	112 (87–128)	<0.001*
Working memory	86 (55–122)	108 (86–139)	<0.001*
Visual memory recognition	93 (62–122)	105 (82–122)	<0.001*
Verbal memory recognition	93 (58–128)	109 (84–128)	<0.001*
Anxiety (BAI)	15 (3–52)	6 (0–34)	<0.001*
Depression (PHQ-9)	6 (0–24)	2 (0–17)	0.002*
Sleepiness (ESS)	10 (0–18)	8 (0–13)	0.016*
Self-efficacy (GSE)	29 (14–40)	34 (22–40)	0.006*
Perceived health status (SF-36v2)			
Subscales			
Physical function	75 (5–100)	100 (15–100)	<0.001*
Role function physical	71 (0–100)	100 (50–100)	<0.001*
Bodily pain	84 (0–100)	84 (41–100)	0.221
General health	68 (10–100)	82 (37–100)	<0.001*
Energy/fatigue	62 (6–100)	63 (18–100)	0.625
Social function	87 (0–100)	100 (25–100)	<0.001*
Emotional function	83 (16–100)	91 (16–100)	0.005*
Mental health	70 (20–100)	80 (35–100)	0.109
Component summary scores			
Physical	49 (27–66)	58 (28–63)	<0.001*
Mental	47 (13–62)	51 (19–64)	0.259

*BAI, Beck anxiety inventory; CHD, congenital heart disease; ESS, Epworth sleepiness scale; GSE, general self-efficacy; PHQ-9, Patient Health Questionnaire Depression Module; SF-36v2, Short-Form-36 Health Survey Version 2; WRAML2, Wide Range Assessment of Memory and Learning, Second Edition*.

*Statistically significant (*p* < 0.05).

The CHD group had 68 and 50% with greater than or equal to mild anxiety and depression symptoms, respectively, compared to 32 and 25% of the healthy controls. Median anxiety and depression scores between CHD and healthy controls were statistically significant (15 vs. 6; *p* = < 0.001 and 6 vs. 2; *p* = 0.002), respectively. Males had more anxiety and depressive symptoms compared to females.

Median sleepiness scores were statistically significant in the CHD group compared to the healthy controls (10 vs. 8; *p* = 0.016), respectively. Excessive sleepiness (abnormal ≥10) was identified in 32% of the CHD group compared to 27% of healthy controls with no gender differences identified.

Median self-efficacy scores were lower in the CHD group compared to healthy controls (28 vs. 34; *p* = 0.006), respectively. Low self-efficacy (scores <30) was identified in 51% of the CHD group compared to 19% in the healthy control group. Lower self-efficacy was associated with worse anxiety, depression, sleepiness, and perceived health status. Males had lower self-efficacy than females in the CHD group.

Perceived health status is summarized in two component scores (physical and mental). Median scores in physical were statistically significant in the CHD group compared to healthy controls (49 vs. 58; *p* < 0.001) with no difference identified in the mental component scores. In the eight subscales, all were statistically significant except for bodily pain, energy/fatigue, and mental health.

### Predictors of Memory Deficits

The list of covariates associated with GMI included in the multivariate model for the total cohort is summarized in Table 4. Gender, number of surgeries, pacemaker, cyanosis at birth, first surgery <30 days of life, number of medications, defect severity, self-efficacy, sleepiness, anxiety, depression, and physical and mental health status were all entered in the stepwise logistic regression if the variables were significant on the bivariate analyses for memory deficits. The final multivariate logistic regression model for GMI is presented in Table 5. Male gender, number of surgeries, anxiety, and self-efficacy were independent predictors which explain approximately 45–64% of the variance for memory deficits using Cox and Snell *R*^2^ or −2 log likelihood, respectively.

**Table 4:** **List of covariates associated with GMI included in the multivariate model**.

Variables	*r*	*p*-Value
Gender	0.18	0.029*
Number of surgeries	−0.60	<0.001**
Pacemaker	−0.29	0.020*
Defect severity	−0.31	0.009**
Infant surgery <30 days	−0.53	<0.001**
Cyanotic at birth	−0.36	0.003*
Number of medications	−0.57	<0.001**
Self-efficacy	0.46	<0.001**
Sleepiness	−0.37	<0.001**
Anxiety	−0.44	<0.001**
Depression	−0.31	<0.001**
Physical health status (SF-36)	0.41	<0.001**
Mental health status (SF-36)	0.18	0.030*

*r = Spearman’s rho correlation coefficient*.

*Correlation is significant at 0.05 level (2-tailed).

**Correlation is significant at 0.01 level (2-tailed).

**Table 5:** **Final multivariate logistic regression model for general memory index (<85 and ≥85)**.

Predictor variables	*B*	SE	*p*	Exp (*B*)	CI (95%) for Exp (*B*)	Model summary
Gender (male)	−1.948	0.781	0.013	0.143	0.031–0.659	Cox and Snell *R*^2^ = 0.45 or −2 log likelihood = 64
Number of surgeries	−1.596	0.327	0.000	0.203	0.107–0.385	
Anxiety	0.079	0.041	0.050	1.082	0.999–1.172	
Self-efficacy	0.326	0.086	0.000	1.385	1.171–1.639	

## Discussion

Significant memory deficits in immediate and delayed tasks were identified in a high proportion of adolescents and young adults with moderate to complex CHD who had undergone surgical palliation at least 10 years previously compared to age- and gender-matched to age- and gender-matched controls. This finding suggests that memory deficits detected at a younger age (13, 34, 35) persists into adolescence and young adulthood. Unintentional or selective “forgetting” is a common behavioral trait seen in adolescence when the information processed is viewed as a low priority or unimportant (36), but in CHD, there could be a behavioral or biologic substrate. Our findings are consistent with three studies by Bellinger et al. (1–3) on young adolescents with complex CHD (d-transposition of the great arteries, tetralogy of Fallot, and single ventricle defects) in which memory was evaluated as part of a neuropsychological assessment and structural brain imaging was performed. In these studies, brain imaging identified focal or multifocal white matter abnormalities but the exact location(s) of injury were not specified in areas affecting memory (1–3). Most covariates in these studies, associated with the GMI scores, were related to the number of surgical or catheterization complications, history of postoperative seizures, and few patient-related factors, such as male gender, birth weight, or gestational age (1–3). Our study similarly identified male gender and number of surgical procedures with additional behavioral-related factors (anxiety and self-efficacy) also emerging as independent predictors of memory deficits. The CHD group was noted to have a higher incidence of ADD/ADHD than the healthy controls. Attentional disorders are more common in males than females and have an increased prevalence in the CHD population (37, 38). Although the incidence of ADD/ADHD was lower in our CHD group compared to other reports (37, 38), this may be the reason why the diagnosis did not emerge as a covariate associated with memory deficits. However, ADD/ADHD is often un-diagnosed and can co-exist with other mood disorders (anxiety/depression) that impact one’s self-efficacy or perceived ability to accomplish the task at hand. Male gender continues to be a risk factor for memory deficits into adolescence and young adulthood with males having worse anxiety and depressive symptom scores than females in our study. This non-modifiable predictor warrants awareness related to gender differences and the need for early assessment, referral, and to engage males in targeted interventions to improve memory deficits.

The multivariate analysis emphasized the importance of patient-related factors compared to clinical or disease specific variables. However, the size and significance of the beta coefficient for the variable number of surgeries suggest that surgery may have a greater impact on memory. The number of surgeries has been reported in other studies to be predictive of abnormal developmental outcomes in younger children (39) or for internalizing (anxiety/depression) and externalizing (attentional disorders) behavior problem in CHD (40). Utens and colleagues (40) also found the number of surgeries to be predictive of behavioral problems and suggested that the number of surgeries, procedures, or hospitalizations could potentially reflect the “experiential” aspect of living with CHD. However, the number of surgeries may also be a surrogate for greater CHD complexity. With advancement in cardiac interventional procedures, such as percutaneous valve replacements, the potential to modify or reduce the total number of surgeries required throughout the lifespan is a realistic possibility for some CHD subgroups (e.g., tetralogy of Fallot) and reduce the risk for procedural focused anxiety.

Our data confirmed that a significant number of adolescents and young adults with CHD had mild to moderate anxiety symptoms compared to controls. Anxiety and depression have been documented previously in adolescents and adults with CHD (41, 42) and can affect cognitive abilities both independently or simultaneously. Ong and colleagues (43) discovered that heart-focused anxiety was associated with parental overprotection and severity of CHD, which may promote feelings of dependency, low self-efficacy, and in turn could potentially compromise cognitive and academic performance. In addition, a positive sense of self, particularly self-efficacy has been associated with better self-care, health status, quality of life, and academic performance (31, 44–46). Anxiety and self-efficacy are modifiable aspects related to psychosocial adjustment in adolescents and young adults living with CHD. Our study findings in the multivariate analysis show anxiety and self-efficacy to have less of an impact on memory deficits compared to other variables (i.e., number or surgeries). Although optimistic, it is unclear the extent that memory deficits will improve with the fostering of psychosocial adjustment.

An interesting discovery in our study was worse verbal compared to visual subtests of the GMI in the CHD group. This finding is consistent with other studies in younger CHD populations in which different verbal memory tasks (e.g., narrative recall, memory of names) were worse than a comparative CHD or control group (34, 35, 45). Some authors suggest this may not be related to memory but to poor attention and alertness and deficiencies in language skills or lower processing speeds needed for verbal memory or executive functioning tasks (34, 35). A study in school age children with hypoplastic left heart syndrome (HLHS) identified deficits in both verbal and visual long-term memory (35). However, visual short-term memory was impaired in children who underwent deep hypothermic circulatory arrest (DHCA) during surgical palliation compared to continued antegrade cerebral perfusion suggesting the impact of DHCA on central nervous system structures involved with visual memory processes. Conversely, Bellinger and colleagues (3) identified better verbal than visual memory for both immediate and delayed trials in adolescents with tetralogy of Fallot without a genetic/phenotypic diagnosis. This finding may be associated with a higher number of complications and surgeries as well as brain image abnormalities in almost half of the study sample which may involve regions related to visual impairment/memory. Our study cohort had significant deficits in attention/concentration which could partially explain worse verbal compared to visual memory. Nonetheless, this finding warrants the use of more visual educational material (e.g., handouts, apps, website material) than verbal training to maintain attention and improved learning/self-care as part of CHD transition/educational programs.

### Clinical Implications for This Study

Our findings are important for providers, patients, and parents to be awareness of potential risk factors associated with memory deficits in adolescents and young adults living with CHD. Unfortunately, certain variables such as male gender and number of surgeries are non-modifiable while anxiety and self-efficacy are amenable to change. More research has suggested that family and maternal factors (i.e., mental health, education, socioeconomic status) may play a more significant role than disease or surgical variables related to behavioral/psychosocial outcomes. However, there are very few reported intervention studies to support psychosocial adjustment in children with CHD and their families (47, 48). Interventions focused on maternal well-being and optimizing parenting skills that focus on self-efficacy and reducing anxiety or worries can be helpful in early childhood development. Other intrinsic or patient factors not included in this study (i.e., hypoxic–ischemic brain injury) that can contribute to memory deficits may benefit from future pharmacological strategies to protect or promote neurogenesis [i.e., thiamine or green tea extract supplementation (49–51) and statins (52)]. Nonetheless, special focus on males with CHD to encourage participation in future interventions is warranted.

Our study should be interpreted in light of some limitations. Although the logistic regression model explained almost half of the variance, other factors not accounted for in this study are making a contribution to memory deficits. Other studies have identified the contribution of maternal factors and socioeconomic status to impact cognitive and neurodevelopmental outcomes (38) which were not measured in this study. However, we did find that the CHD group had a higher prevalence of public vs. private insurance compared to controls. Parents suspicious of a memory problem in their child might have been more motivated to participate, whereas parents of children who perform well at school or parents not wanting to potentially find another problem with their child may have decline disproportionately to participate. Our sample was heterogeneous with the majority of CHD diagnoses classified as moderate to severe and cannot be generalized to simple/less complex forms of CHD. The sample size prohibited further group comparison related to defect severity. The numbers of participants with genetic syndromes or ADD/ADHD in both groups may be higher as testing was not performed and only identified *via* medical chart review in CHD participants or parental self-report in healthy controls. Primary care chart reviews were not performed on healthy controls as this would be difficult or almost impossible due to inconsistent medical follow-up during adolescents creating potential sample bias. Generalizability of our findings can be challenging given the high proportion of Hispanic participants which is quite different from other cardiac centers. Furthermore, most CHD participants had their last surgical procedure over a decade ago and did not have the advantages in improved surgical technique and medical management of the current era. Neuroimaging was not performed for this study, so the incidence of congenital or acquired brain injury is unknown. However, neuroimaging is currently being performed by our research team in the single ventricle participants in relation to brain structures that affect memory, and we are hopeful that this will provide clarification on the relationships between brain structure and cognitive status in these subjects.

## Conclusion

A combination of fixed and modifiable factors influenced memory deficits in adolescents and young adults with moderate to complex CHD after surgical palliation. In this cohort of adolescents and young adults, more patient-related (self-efficacy, anxiety, male gender) rather than clinical factors (number of surgeries) were predictors of memory deficits. Deficits emerged in verbal memory, attention/concentration, working memory, and memory recognition compared to healthy control. However, visual memory appeared to be less affected. Therefore, to potentially enhance adolescent CHD self-care, clinicians should explore the development of clinical interventions targeted to reduce anxiety, improve self-efficacy, and increase use of visual patient education material in transition educational programs in the CHD population.

## Author Contributions

NP: study concept/design, data analysis, interpretation, and drafting of the original manuscript. WE and DF: acquisition of data. MW, MP, NH, AL, and RK: analysis and interpretation of the data and critical revisions of the manuscript. All the authors approved the final version of the manuscript.

## Conflict of Interest Statement

The authors declare that the research was conducted in the absence of any commercial or financial relationships that could be construed as a potential conflict of interest.

## Abbreviations

ADD, attention deficit disorder; ADHD, attention deficit hyperactivity disorder; BAI, Beck anxiety inventory; CHD, congenital heart disease; CPB, cardiopulmonary bypass; DHCA, deep hypothermic circulatory arrest; ESS, Epworth sleepiness scale; GMI, general memory index; GSE, general self-efficacy; HLHS, hypoplastic left heart syndrome; PHQ-9, Patient Health Questionnaire Depression Module; SF-36v2, Short-Form-36 Health Survey Version 2; WRAML2, wide range of assessment memory and learning 2nd edition.
